# Explainable prediction model for the human papillomavirus status in patients with oropharyngeal squamous cell carcinoma using CNN on CT images

**DOI:** 10.1038/s41598-024-65240-9

**Published:** 2024-06-20

**Authors:** Annarita Fanizzi, Maria Colomba Comes, Samantha Bove, Elisa Cavalera, Paola de Franco, Alessia Di Rito, Angelo Errico, Marco Lioce, Francesca Pati, Maurizio Portaluri, Concetta Saponaro, Giovanni Scognamillo, Ippolito Troiano, Michele Troiano, Francesco Alfredo Zito, Raffaella Massafra

**Affiliations:** 1 Laboratorio Biostatistica e Bioinformatica, I.R.C.C.S. Istituto Tumori ‘Giovanni Paolo II’, Bari, Italy; 2 Radiation Oncology Unit, Dipartimento di Oncoematologia, Ospedale Vito Fazzi, Lecce, Italy; 3 Ospedale Monsignor Raffaele Dimiccoli, Barletta, Italy; 4 Unità Operativa Complessa di Radioterpia, I.R.C.C.S. Istituto Tumori ‘Giovanni Paolo II’, Bari, Italy; 5 Radiotherapy Department, ASL Brindisi, Brindisi, Italy; 6 Unità Operativa Complessi di Anatomia Patologia, I.R.C.C.S. Istituto Tumori ‘Giovanni Paolo II’, Bari, Italy; 7 Radiation Oncology Department, Fondazione IRCCS “Casa Sollievo della Sofferenza”, San Giovanni Rotondo, Italy

**Keywords:** Human papillomavirus, Oropharyngeal squamous cell carcinoma, Convolutional neural network, Explainable artificial intelligence, Grad-CAM, Cancer imaging, Head and neck cancer

## Abstract

Several studies have emphasised how positive and negative human papillomavirus (HPV+  and HPV−, respectively) oropharyngeal squamous cell carcinoma (OPSCC) has distinct molecular profiles, tumor characteristics, and disease outcomes. Different radiomics-based prediction models have been proposed, by also using innovative techniques such as Convolutional Neural Networks (CNNs). Although some of these models reached encouraging predictive performances, there evidence explaining the role of radiomic features in achieving a specific outcome is scarce. In this paper, we propose some preliminary results related to an explainable CNN-based model to predict HPV status in OPSCC patients. We extracted the Gross Tumor Volume (GTV) of pre-treatment CT images related to 499 patients (356 HPV+ and 143 HPV−) included into the OPC-Radiomics public dataset to train an end-to-end Inception-V3 CNN architecture. We also collected a multicentric dataset consisting of 92 patients (43 HPV+ , 49 HPV−), which was employed as an independent test set. Finally, we applied Gradient-weighted Class Activation Mapping (Grad-CAM) technique to highlight the most informative areas with respect to the predicted outcome. The proposed model reached an AUC value of 73.50% on the independent test. As a result of the Grad-CAM algorithm, the most informative areas related to the correctly classified HPV+ patients were located into the intratumoral area. Conversely, the most important areas referred to the tumor edges. Finally, since the proposed model provided additional information with respect to the accuracy of the classification given by the visualization of the areas of greatest interest for predictive purposes for each case examined, it could contribute to increase confidence in using computer-based predictive models in the actual clinical practice.

## Introduction

In the last decade, the incidence of oropharyngeal squamous cell carcinoma (OPSCC) showed an increase if compared to other head and neck cancers, and animportant percentage of OPSCCs are related to human papillomavirus (HPV) infections^1,2^. The latest edition of the American Joint Committee on Cancer (AJCC) staging system emphasised how positive HPV(HPV +) and negative HPV(HPV–) OPSCCs have distinct molecular profiles, tumor characteristics, and disease outcomes^3^. Since it has been demonstrated how HPV+ patients have a favourable prognosis and a better response to radio-chemotherapy^4–6^, the assessment of the HPV status in OPSCC patients is a key factor for defining a personalised therapeutic plan. In OPSCC patients, HPV status is routinely assessed on biopsies by immunohistochemistry of the p16 expression level^7^. However, this is an expensive procedure that can be performed only in equipped and specialised laboratories, which could be not available in all the hospitals. Hence, less expensive, and invasive support tools that could replace the immunohistochemical examination of the p16 expression level are required. As proof of concept, some efforts have been freshly made in defining radiomic-based models to predict HPV status by exploiting radiological exams, such as MRI, PET, CT^8–14^. Among them, CT exams were the most widely used for the development of automated models aiming at HPV prediction. These studies demonstrated a significant association of radiomic features with the HPV status^15–20^. The proposed models based on CT images have shown encouraging results, although less performing than other proposals developed starting from second-level radiological exams, such as MRI and PET. However, there is a lack of evidence explaining the role of radiomic features in achieving specific outcomes. Indeed, the Artificial Intelligence (AI) algorithms used to extract radiomic features and to perform a prediction are often based on complex mathematical underpinnings, thus remaining black-boxes. The scepticism of clinicians in the use of AI-tools is connected to the concern that the decision-making process of these black boxes could be biassed and may lead to severe repercussions in clinical practice. This concern is even more amplified when deep learning techniques, such as Convolutional Neural Networks (CNNs), are used^21–23^. Recently, eXplainable Artificial Intelligence (XAI), whose main aim is to overcome the black-box concept and define more intelligible tools to be used in clinical practice in a more informed manner^24,25^, was introduced**. **Within this emerging scenario, in this paper, we present some preliminary results related to an explainable HPV status prediction model in OPCSS patients. Specifically, a well-known CNN architecture was trained from scratch by using CT images from a public database and then validated on an independent multicentric database. An XAI paradigm was applied to the final result to identify the areas within the image under study that had the greatest influence on the definition of the final classification result.

## Materials and methods

### Experimental dataset

For this study, we used two datasets. The first dataset was the OPC-Radiomics^26,27^ head and neck cancer collections of the publicly accessible TCIA^28^. For each patient, the public dataset provided pre-treatment CT image and HPV status tested by immunohistochemical (IHC)-based p16 staining. The OPC-Radiomics dataset consisted of 608 cases, but only 499 were eligible for the study according to the following inclusion criteria: histologic diagnosis of squamous cell carcinoma of the oropharynx (OPSCC), availability of pre-treatment CT, and HPV status detected by immunohistochemical (IHC)-based p16 staining. Among the 499 enrolled patients, 356 were HPV+. The second dataset was a multicentric dataset, which was employed as an independent test set. We enrolled 92 patients across multiple centres in Apulia region (Italy), out of which 13 patients were referred to Istituto Tumori “Giovanni Paolo II” in Bari (Apulia, Italy), 20 patients to Casa Sollievo della Sofferenza Hospital in San Giovanni Rotondo (Apulia, Italy), 40 patients to “Vito Fazzi” Hospital in Lecce (Apulia, Italy), 15 patients to Perrino Hospital in Brindisi (Apulia, Italy), and 4 patients to “Monsignor Raffaele Dimiccoli” Hospital in Barletta (Apulia, Italy). From January 2015 to December 2022, all patients were consecutively included in a data registration program as part of routine clinical practice. Patients were enrolled according to the inclusion criteria mentioned above. Among the 92 enrolled patients, 43 were HPV+. Pre-treatment CT was used for contouring (after imaging fusion with neck enhanced MR or neck enhanced CT) and for radiotherapy planning. We analysed the images without contrast medium, which were used for contouring and radiotherapy planning. All the exams were acquired using dedicated and customized immobilization and reproducibility systems (SIRs) (versa board and 9-point thermoplastic mask). The corresponding series were generated by an area subtended between the keel bifurcation and the vertex of the head, by using an acquisition spiral with a thickness of 3 mm with pitch equal to 1 (contiguous scans), 120 kV, and 350 mAs. The field of view (FOV) used was the maximum one (600 mm) with a standard brain acquisition filter and a 512 × 512 matrix. Table 1 summarises the main characteristics of both datasets. This study was approved by the Ethic Committee of Istituto Tumori “Giovanni Paolo II” Bari, Italy (Prot. n. 1170/CE). All the institutes involved in the study signed a data transfer agreement.

**Table 1:** Ptient characteristics of both OPC-Radiomics and multicentric datasets.

Characteristics	Distribution (%)	
	OPC-Radiomics	Multicenter dataset
HPV status		
Positive (Abs.; %)	356; 71.34%	43; 46.74%
Negative (Abs.; %)	143; 28.66%	49; 53.26%
Age at diagnosis		
Median (1th-3th quartiles)	59.88 (53.82–67.49)	62.00 (55.00–69.25)
Gender		
Female (Abs.; %)	105; 21.04%	19; 20.65%
Male (Abs.; %)	394; 78.96%	73; 79.35%
Tumor size classification		
T1 (Abs.; %)	90; 18.04%	12; 13.04%
T2 (Abs.; %)	162; 32.46%	39; 42.39%
T3 (Abs.; %)	147; 29.46%	20; 21.74%
T4 (Abs.; %)	100; 20.04%	21; 22.83%
Lymph Node classification		
N0 (Abs.; %)	83; 16.63%	12; 13.04%
N1 (Abs.; %)	48; 9.62%	19; 20.65%
N2 (Abs.; %)	326; 65.33%	48; 52.17%
N3 (Abs.; %)	42; 8.42%	13; 14.13%
ECOG		
0 (Abs.; %)	323; 64.73%	37; 40.22%
1 (Abs.; %)	125; 25.05%	48; 52.17%
> 1 (Abs.; %)	49; 9.82%	7; 7.61%
Nan (Abs.; %)	2; 0.40%	–

### Image analysis by convolutional neural network model

Referring to the multicentric dataset, for each patient, gross primary tumor volume (GTV) was manually contoured for treatment planning purposes. The delineation was not standardised. It was performed according to clinical protocols, which were different for each institution. Moreover, all the cases belonging to the public dataset had segmentation of GTVs at disposal. Firstly, for each patient, the CT slice, which included the largest GTV, was selected. The GTV region was then automatically segmented by extracting a binary mask for the structures of interest. Specifically, from the GTV contouring performed by the radiologist on the pre-treatment CT, a binary mask was extracted. The binary mask of the GTV was then used to crop the region of interest (ROI) on the starting CT image. The resultingROI was normalized in the range [0;1] and the image intensity values were adjusted by suturing the bottom 1% and the top 1% of all pixel values.

**Figure 1:**
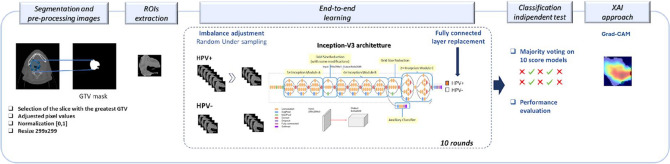
Workflow of the proposed model.

A CNN architecture was trained from scratch (end-to-end training) to solve the classification problem (HPV+ cases versus HPV− cases) starting from the ROI of each patient (Fig. 1). In the deep learning paradigm, the end-to-end framework means that the network performs both feature extraction and classification processes. Specifically, we used Inception-v3 architecture^29^, that is a CNN with 48 layers that bring several improvements over previous versions.

The network required an input image size of 299-by-299 pixels, hence, each ROI was resized. Since we solved a binary classification task (HPV+ *versus* HPV−), the final fully connected layer was replaced with a 2-class output. The model was trained over 100 epochs with an initial learning rate of 10–4, batch size equal to 12 elements, L2 regularization factor equal to 10–4, and Adam optimizer^30^. Moreover, the shuffling of the training and validation data before each training epoch was performed.

The network was trained on the OPC-Radiomics dataset and subsequently validated on the multicentric dataset (independent test set). Since the OPC Radio-mics data set was highly unbalanced, a well-known under sampling balancing technique was applied. It consists in randomly selecting a sub-sample of the class with the largest number of samples. In our case, we selected a sub-sample with the same number of the class with a smaller number of patients. We iterated the random subsampling for ten times and trained the network on each of these balanced subsamples. For each trained model, the optimal threshold on the probability scores generated by the network was computed by using Youden's index on the Receiver Operating Characteristic (ROC) curve^31^. For the sake of the robustness of our analysis, we implemented a data augmentation process using four transformations, such as Random Translation, Random Reflection, Random Rotation, and Random Scale^32^.

The ten models trained on balanced subsamples of the training setwere applied to the independent test set. The majority voting technique was applied on the binary result returned by the ten models. Then, in each case of the test sample, either the highest or smallest classification score was associated when the predicted class by majority voting technique was HPV+ or HPV−, respectively.

Finally, we applied Gradient-weighted Class Activation Mapping (Grad-CAM) interpretability technique^33,34^ to highlight the zones of the analysed images that contribute to the decision of the implemented CNN. The Grad-CAM algorithm performs the gradients of the classification score with respect to the final convolutional feature map. It is a generalisation of the class activation mapping (CAM) technique. Unlike the CAM algorithm, it produces visual explanations without re-training the model architecture.

Classification performances of the CNN architecture were evaluated in terms of Area Under the Curve (AUC), and standard metrics, such as Accuracy, Sensitivity, and Specificity, which were calculated after identifying the optimal threshold using Youden’s index on the ROC curves in each round^31^. All the analyses were performed by using MATLAB R2022a (MathWorks, Inc., Natick, MA, USA) software.

### Ethical approval

The study was conducted according to the guidelines of the Declaration of Helsinki and approved by Ethics Committee of Istituto Tumori “Giovanni Paolo II,” Bari, Deliberation n.138/2023.

## Results

### Classification performances

In this work, we proposed an end-to-end training of Inception-V3 CNN architecture by providing in input the ROIs extracted from the CT images of the public database, which consisted of 499 patients (143 HPV−). Since the training dataset was unbalanced, the random under sampling technique was applied for 10 times. Then, ten models were trained on the balanced sub-samples consisting of 284 patients.

The trained models were then used to predict HPV status on the independent test set (92 patients, out of which 43 HPV+ cases). As described above, a majority voting technique was applied to obtain the final classification outcome.

**Figure 2:**
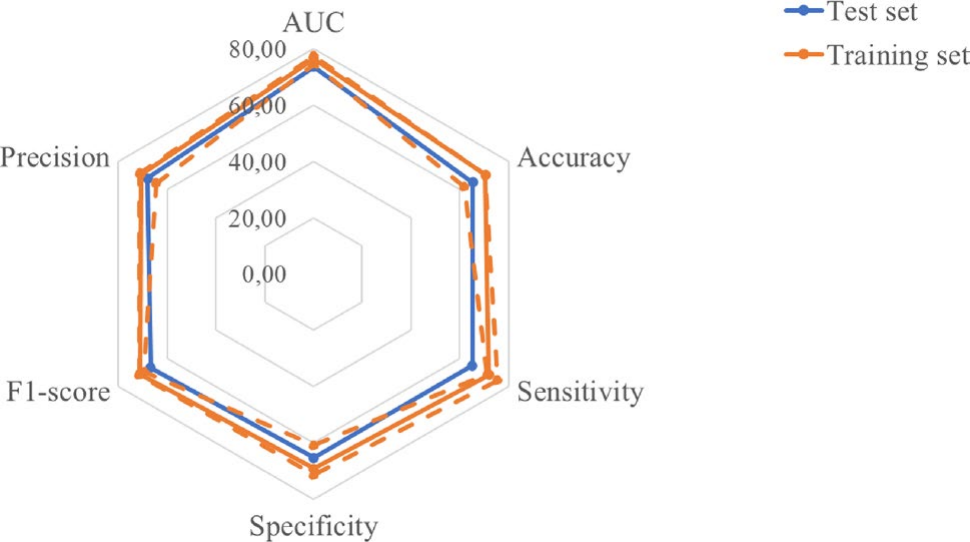
Performance classification on training and test sets.

Figure 2 shows the classification performances obtained on both the training and the independent test sets, respectively. The model trained on the training set reached a median AUC and accuracy values of 76.40% (74.52-77.43, 1st-3rd quartiles) and 70.42% (61.73-70.43, 1st-3rd quartiles), respectively. In particular, the model reached on training set a sensitivity, specificity, precision, and F1-score of 71.83% (71.13%-75.35%), 69.01% (60.82%-71.31%), 70.86% (64.55%-71.33%), and 71.24% (68.73%-73.28%).

On the independent test, the AUC and accuracy values were 73.50% and 65.22%, respectively, with a sensitivity, specificity, precision, and F1-score of 65.12%, 65.31%, 68.09%, and 66.67%, respectively.

### Activation map on independent test set obtained by Grad-CAMP algorithm

An XAI algorithm was applied to evaluate the areas within the ROI that most contributed to the achievement of the classification score and the resulting predicted class. Although the performance of the model was not particularly efficient and requires an optimization phase, it was possible to underline some insights.

**Figure 3:**
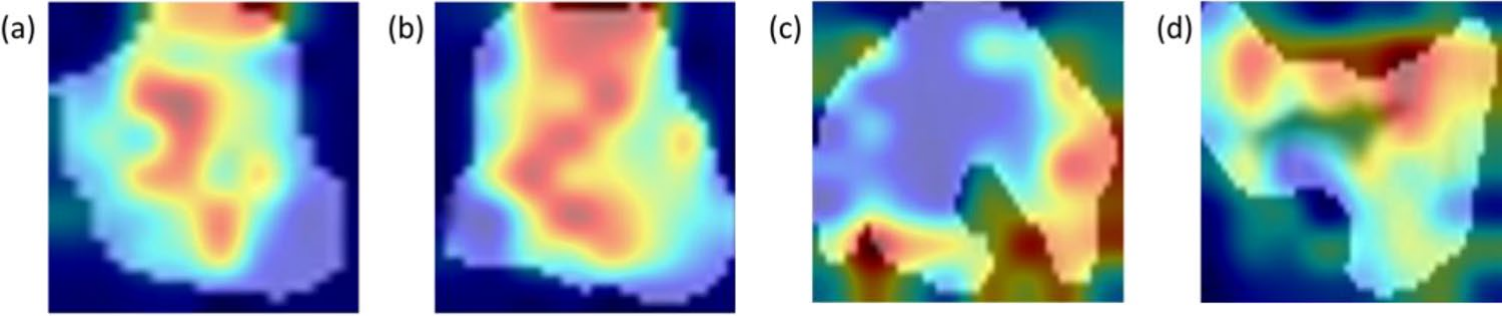
Examples of activation maps referring to correctly classified patients generated by the Grad-CAM algorithm. The first two images refer to two HPV+  cases, while the last two images refer to two HPV− cases. The red areas are the regions that have most influenced the process of assigning the positive or negative class.

Figure 3 shows some examples referring to cases of patients from the validation dataset correctly classified by the model. The red areas, i.e., the most involved in the decision-making process of the classifier, were those both within the image and on the edges. With reference to correctly classified HPV+ patients, the most informative areas involved the intratumoral areas (Fig. 3a,b). On the contrary, with reference to correctly classified HPV− patients, the areas most involved seemed to be concentrated on the edges (Fig. 3c,d).

**Figure 4:**
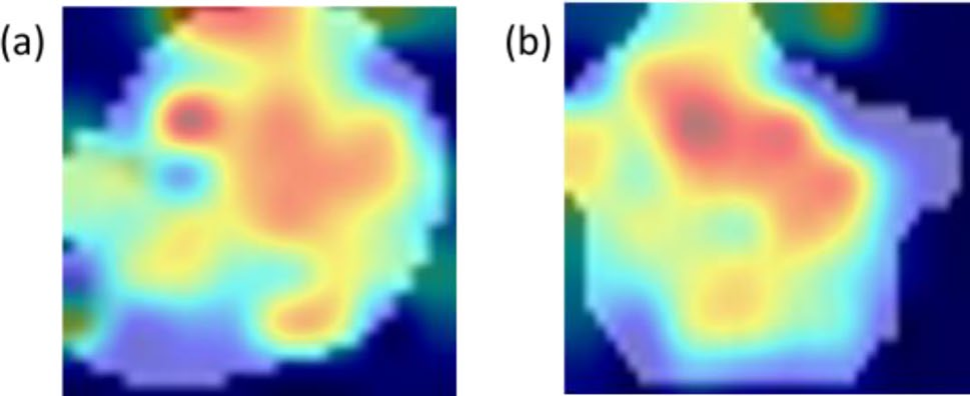
Examples of activation maps for misclassified patients generated by the Grad-CAM algorithm. The first image refers to a real HPV+  case, while the second to a real HPV− case. The red zones are the regions that have most influenced the positive or negative class assignment process.

Figure 4 shows two examples of incorrect classification. For an HPV+ patient erroneously classified by the system as an HPV− case, the areas that mostly contributed to the attribution of the negative class also involved some edges (Fig. 4a). On the contrary, for an HPV− patient erroneously attributed as an HPV+ case, the algorithm based its decision on internal areas alone (Fig. 4b).

## Discussion and conclusion

Epidemiological studies show an increase in the incidence of HPV-associated OPSCC^35,36^. The detection of HPV status is becoming an essential diagnostic factor in defining the optimal path of care for patients. Indeed, recent studies show that HPV+ patients have a more favourable prognosis and are more radiosensitive compared with HPV− patients^37^. HPV status determination by immunohistochemical examination of p16 expression level is the most frequently used, due to its lower cost compared with other laboratory tests which are currently available in clinical practice. However, this is an examination that requires equipped laboratories that may perform these analyses with non-negligible costs for the healthcare system. Therefore, the prediction of HPV status based on the analysis of biomedical images commonly performed in clinical practice, such as the mandatory CT examination for tumor staging, is currently a task of great interest in the scientific community.

Innovative AI techniques applied to biomedical image analysis are providing exceptional performances in the main diagnostic and prognostic tasks. Nevertheless, the adoption of clinical practice support tools developed with techniques fail to find a real application in the clinical practice. One of the main obstacles is represented by the fact that AI algorithms are often considered as black-boxes. This is emphasised by the use of deep learning algorithms, which are more complicated than machine learning algorithms for their inner interconnected structures and millions of trainable parameters. Indeed, the systems proposed in the state-of-the-art very often do not provide information on why and how the model has made a given prediction. In this context, XAI techniques have recently been introduced to understand how a predictive model reached a given decision.

With reference to the focus of this work, there are several studies using AI techniques on CT images^15–20^. Most of these works are based on handcrafted features to train well-known state-of-the-art classifiers and, although they often need to be validated on larger cohorts, the potential performances present high quality^15,17,18,20^. Only recently, pioneering works for predicting HPV status by applying CNNs have been proposed^17,19^. However, the analysed cohorts were heterogeneous in terms of the assessment of the real HPV status, not including p16 expression level only.

To the best of our knowledge, XAI techniques within this research field have not been implemented yet.

In this paper, a preliminary XAI model exploiting pre-treatment CT images for the prediction of HPV status in OPCSS patients was proposed. We trained an end-to-end Inception-V3 architecture on OPC-radiomics public database. This dataset consisted of patients with OPCCS for whom HPV status had been assessed by immunohistochemical examination of p16 expression level. Then, the learned network was tested on a multicentric independent dataset, and for this data set, HPV status was assessed using the same procedure of training dataset.

Since head and neck CT scans are highly susceptible to dental implant artifacts, we used ROIs containing the tumor. On the independent test set, the proposed predictive model achieved a median AUC and accuracy values of 73.50% and 65.22%, respectively. Although the results are encouraging, these findings are currently not applicable into clinical practice.

Compared to other models based on radiomic features extracted from CT images, the performances achieved were just about 8 percentage points lower. However, it should be emphasised that these models were trained on very small data sets^17,18,20^ or on heterogeneous datasets with respect to the HPV status assessment method^16,19^. With respect to this last aspect, it should be underlined that the p16 test is highly sensitive, but less specific than the HPV test evaluated with other molecular methods^38,39^. Therefore, the performance of our model could be underestimated to the extent that the system true positives were not really positive. Moreover, our performance can also be compromised by possible artefacts present in the public database which, as demonstrated in the state-of-the-art, may significantly reduce the accuracy of the model^15^.

The implemented XAI approach allows us to visualise the areas of interest that have contributed to the assignment of one class rather than the other one. In general, from our preliminary results, the areas of greatest interest appear to be those inside the GTV and at the edges.

According to some recent works, there are qualitative morphological characteristics deducible from radiological images that distinguish HPV+ from HPV− tumors^8,40–42^: HPV− tumors tend to have an irregular shape and a greater intratumoral heterogeneity than HPV+ tumors. In accordance with these findings, the areas of greatest interest for the implemented XAI technique mainly included both the intratumoral area and the edges. This information, in agreement with the indications provided by expert radiologists in cutting-edge works, contributes to increasing confidence in the potential of the proposed tool. Indeed, the activation maps visually show which characteristics and areas have been considered in the decision-making process and allow the clinician to evaluate the appropriateness of the result provided with respect to the qualitative judgement.

The main limitation of the proposed work is related to the sample size that can be overcome by validating the model on larger cohorts. Furthermore, since the soft tissue resolution of CT was not high, determining the boundary of oropharyngeal cancer lesions was challenging. In our proposed model, the accurate delineation of the ROI could directly affect the accuracy of the final model. Despite in our previous work—whose purpose was to predict possible radio-induced toxicities in patients with oropharyngeal cancer^43^—we demonstrated how the AI-model was robust to variations in ROI contouring, current model could be optimized considering different ROIs (for example peritumoral region) or considering 3D tumor volume, in order to reduce the problems related to artifacts.

Finally, in this preliminary model we have only normalised the CT images provided by from different centres. However, since the validation dataset was multicentre, future studies will concern the implementation of more sophisticated CT image harmonization techniques and the evaluation of any resulting improvement in classification performance.

Finally, we aim to underline the innovativeness of the proposed approach to the task to solve. Providing additional information with respect to classification accuracy could aid clinicians to increase their confidence in the suggestion proposed 'by the machine'.

## Data Availability

The data was obtained from the open-access OPC-Radiomics dataset publicly available at The Cancer Imaging Archive (TCIA) database (https://wiki.cancerimagingarchive.net/pages/viewpage.action?pageId=70226325). Moreover, the raw data supporting the conclusions of this article will be made available by the corresponding authors, without undue reservation. Because of ethical reasons, the author-generated code is available upon request. The code request may be sent to the scientific direction (e-mail: dirscientifica@oncologico.bari.it).
